# Perceived factors shaping unsafe abortion practices among young women in Bongo District, Ghana

**DOI:** 10.3389/frph.2026.1926198

**Published:** 2026-07-22

**Authors:** Joel Afram Saah, Richard Opoku Asare

**Affiliations:** 1 Faculty of Health and Allied Sciences, Regentropfen University College, Bolgatanga, Upper East Region, Ghana; 2 International Educated Nurses, Toronto, ON, Canada

**Keywords:** Bongo District, contraceptive use, Ghana, unsafe abortion, young women

## Abstract

**Background:**

Unsafe abortion remains a critical public health challenge among young women in Ghana, persisting despite the relatively liberal provisions of PNDC Law 102.

**Aim:**

This qualitative study explored perceived factors associated with unsafe abortion among women aged 15–25 years in the Bongo District, Upper East Region, focusing on contraceptive knowledge and use, health-system access, and socio-cultural determinants.

**Methods:**

Thirty-four women from five communities in Bongo District were recruited through purposive sampling and interviewed using a semi-structured guide. Data were analysed thematically using Braun and Clarke's six-phase framework.

**Results:**

Four themes were identified. First, participants perceived unsafe abortion to be common in the district. Second, contraceptive non-use was attributed less to a lack of awareness of methods than to fear of side effects, infertility, and partner disapproval. Third, formal health services were perceived as expensive, distant, and staffed by judgmental providers, which discouraged their use. Fourth, fear of family shame and expulsion from school led many young women toward cheaper, more discreet informal providers.

**Conclusion:**

Participants’ accounts suggest that unsafe abortion in Bongo District reflects structural gaps in the reproductive health system, weak social support, and gaps in policy implementation, rather than a lack of individual knowledge. Findings indicate that young women in the district are generally aware of the risks of unsafe procedures but perceive themselves as having limited alternatives within their socio-cultural and religious context.

## Introduction

Unsafe abortion is a major, largely preventable contributor to maternal morbidity and mortality in low- and middle-income countries (LMICs) (1). The World Health Organization and the Guttmacher Institute estimate that nearly 45% of abortions worldwide are unsafe, that 97% of these occur in developing countries, and that unsafe abortion accounts for an estimated 4.7–13.2% of maternal deaths globally (2, 3). Sub-Saharan Africa carries a disproportionate share of this burden, reflecting a combination of restrictive or poorly implemented laws, under-resourced health systems, and strong social stigma surrounding abortion (2).

Ghana has one of the more liberal abortion laws in sub-Saharan Africa: the Criminal Code (Amendment) Law 1985, PNDC Law 102, permits abortion under defined circumstances, including sexual assault, incest, or danger to the woman's life or health. Despite this, unsafe abortion is estimated to contribute to around 11% of maternal deaths nationally (4). Persistent stigma, fear of discrimination, and limited public awareness of the law's provisions mean that many women, particularly in rural areas, do not access safe services even where these are technically permitted. Young women aged 15–25 years are especially affected, given their comparatively limited contraceptive knowledge and access, continued exposure to abortion-related stigma, and socio-economic constraints (5).

District-level evidence on these dynamics remains limited, particularly for the Bongo District in the Upper East Region, an area characterised by distinct sociodemographic conditions, a thin health infrastructure, and persistent inequalities in sexual and reproductive health (SRH) outcomes. The district's formal health system comprises 28 facilities, including one district hospital, six health centres, 21 Community-Based Health Planning and Services (CHPS) compounds, and three private clinics; however, 17 of the 21 CHPS compounds lack permanent structures, limiting the privacy and reliability of consultations available to residents. Service fees present a further barrier for many young women, most of whom are unemployed, and a substantial informal sector of traditional healers and chemical sellers has emerged, valued for being more accessible, affordable, and discreet than formal facilities. Deep-seated socio-cultural and religious norms compound these structural constraints: stigma attached to unmarried pregnancy leads many young women to prioritise secrecy over safety, and providers themselves are sometimes reluctant to offer abortion-related services because of anticipated community backlash or misunderstanding of what the law permits (6, 7).

District-specific evidence is important both because abortion incidence and safety outcomes vary considerably across Ghana, and because effective interventions must be tailored to local health-system and socio-cultural conditions. National and regional statistics cannot capture the complex interplay of individual, health-system, and socio-cultural factors that shape a young woman's decision to seek care from a formal facility or an informal provider. This study addresses this gap by exploring, through the lens of the Health Belief Model, how young women aged 15–25 years in Bongo District perceive and describe the factors shaping unsafe abortion practices. The aim is to generate locally grounded evidence to inform youth-focused reproductive health programming, family planning outreach, and district-level health investment. Specifically, the study sought to:

1. Explore young women's perceptions and lived experiences of unsafe abortion in Bongo District.
2. Describe perceived barriers to contraceptive knowledge and use in relation to unsafe abortion.
3. Identify perceived health-system and structural barriers to safe abortion care among young women in the district.

## Methods and materials

### Research design

This study adopted an exploratory, descriptive qualitative design (8), chosen over more formalised approaches such as phenomenology or grounded theory because the aim was to describe and understand the social, cultural, and structural context of unsafe abortion among young women in Bongo District rather than to generate new theory. This approach is well suited to research questions that are context-specific, pragmatic, and programme-oriented.

A qualitative approach was appropriate because the research questions concern abortion decision-making in a setting where the topic is stigmatised, sensitive, and shaped by unequal power relations. Issues of disclosure, fear, community surveillance, and gendered power dynamics are unlikely to be adequately captured through structured surveys. In-depth qualitative interviewing allowed patterns of norms, beliefs, and behaviour to emerge in participants’ own words, and enabled the researchers to situate individual accounts within their broader social context (9).

### Study area

Bongo District is situated in the Upper East Region of Ghana, covers 459.5 square kilometres, and shares its northern border with Burkina Faso. As noted above, the district's 28 health facilities include one district hospital (Bongo District Hospital, the main referral centre for comprehensive and post-abortion care), six health centres, 21 CHPS compounds, and three private clinics (Ayamfooya Memorial Clinic, Kongo; Abitanga Medical Centre, Soe; and Aloobo International General Clinic, Bongo-Namoo). The limited privacy of many CHPS compounds and the cost of formal services, set against the accessibility, affordability, and discretion of informal providers, form an important backdrop to the findings reported below.

### Study population

The study population comprised women aged 15–25 years living in Bongo District, an age range associated with heightened vulnerability to unintended pregnancy and unsafe abortion, alongside declining reproductive autonomy, lower family-planning use, limited health literacy, stigma, and partner pressure (10). According to the 2021 Population and Housing Census, Bongo District has a total population of 120,254, of whom women constitute 52.7% (*n* = 63,334). To capture variation in geographic remoteness and service availability, participants were recruited from five communities: Bongo Central, Soe, Namoo, Gowrie, and Kunkua.

### Recruitment procedure

Participants were identified and approached with the assistance of community health volunteers, female peer leaders, and CHPS outreach workers who were already known and trusted within each community. These intermediaries first introduced the study informally to potentially eligible young women in private or semi-private settings, such as their homes or other locations away from public view, without disclosing the identity of other participants or details of individual cases. Women who expressed interest were then invited to a private location of their choosing for a fuller explanation of the study and, where appropriate, to complete the consent or parental/guardian consent and assent process described below. No recruitment activity took place in public spaces such as health facility waiting areas, and community health volunteers were briefed on the sensitivity of the topic and instructed not to discuss any individual's participation with others.

### Inclusion and exclusion criteria

#### Inclusion criteria

Participants were eligible if they: (i) were female, aged 15–25 years at the time of data collection; (ii) were permanent or long-term residents of Bongo District; (iii) were able to provide voluntary informed consent, or, if under 18 years, informed assent alongside parental or guardian consent; (iv) were able to communicate in English, Gurune (Frafra), or another local language with the assistance of an interpreter; and (v) had knowledge of unsafe abortion in the community, whether through direct personal experience, the experience of a close social contact, or community-level observation.

Because these represent different forms of knowledge with different evidentiary weight, participants were also asked, as part of the interview, to characterise the basis of their knowledge. Of the 34 participants, 1 had direct personal experience of unsafe abortion, 13 described the experience of a close social contact, and 20 spoke primarily from community-level observation. This distinction is considered in the interpretation of the findings below, and direct personal accounts are identified as such where relevant.

#### Exclusion criteria

Participants were excluded if they: (i) were below 15 or above 25 years of age; (ii) were not residing in Bongo District at the time of data collection; (iii) exhibited visible acute physical or psychological distress at recruitment; (iv) were unwilling or unable to provide informed consent; or (v) had already participated in a prior data collection session for this study.

### Sampling strategy

A purposive sampling method was used, consistent with qualitative research principles that prioritise information-rich cases over statistical representativeness. Participant selection was guided by four criteria: being female; aged 15–25 years and resident in Bongo District at the time of interview; and having direct or indirect knowledge of unsafe abortion in the community, ensuring that all participants could speak to the study's three thematic areas of interest. This sampling approach is consistent with the study's theoretical framework: because the Health Belief Model is concerned with how individuals perceive risk and make health decisions within specific social contexts, selecting participants embedded in those contexts ensured coherence between the sample, methods, and analytical framework.

### Sample size and data saturation

Sample size was determined by data saturation, the point at which further interviews no longer generated new thematic content. Recruitment continued across all five communities until saturation was reached across five thematic areas: perceived prevalence; motivations for seeking abortion; contraceptive knowledge and use; health-system access; and socio-cultural and economic determinants. Saturation was achieved at 34 participants, within the range of 20–40 typically recommended for thematic analysis of semi-structured interview data (11).

### Research instrument

Data were collected using a semi-structured interview guide comprising five sections: demographic information; experiences of and attitudes toward unsafe abortion; contraceptive knowledge and barriers; health-system access; and socio-cultural and economic determinants. The guide fixed a common set of topics while allowing follow-up on issues raised spontaneously, an approach suited to sensitive topics such as anticipated stigma from health providers or fear of being identified within the community. The tool was reviewed by the research supervisor for content validity and pilot-tested with young women in a nearby community; minor revisions followed, including simplifying the phrasing of some questions to improve comprehension among participants with lower levels of formal education.

### Data collection procedure

Data were collected through individual, in-person interviews. Before each interview, participants were informed of the study's purpose, the nature of the questions, their right to decline any question or withdraw at any time without penalty, and the measures in place to protect their confidentiality. Informed consent (and, for minors, assent alongside parental or guardian consent) was obtained both verbally and in writing.

Interviews took place in community centres, quiet outdoor spaces, or participants’ homes, selected to maximise privacy given the stigma surrounding the topic. Participants were reassured that there were no “correct” answers and that the purpose was to understand their experiences rather than to evaluate them. Where Gurune (Frafra) was the participant's preferred language, a trained female research assistant fluent in Gurune and English provided interpretation. Responses were recorded in writing and, with participants’ permission, by audio recorder, and all recordings were transcribed verbatim into English. Data collection continued until saturation was reached across all five thematic areas.

At the close of each interview, participants were given information about, and where relevant referred to, existing reproductive health, counselling, and post-abortion care services available through the district health system, in case the interview raised distress or revealed an ongoing need for care.

### Ethical considerations for minors

Because the study included participants aged 15–17 years, additional safeguards were applied beyond standard procedures. Recruitment and consent discussions with younger participants took place in private settings away from parents, peers, and other community members to reduce the risk that participation, or its subject matter, would become known within the household or wider community. Parental or guardian consent was sought only after the minor had indicated willingness to participate, and interviewers were trained to end or pause an interview if a minor showed signs of distress. Interview data from minors were stored and anonymised using the same procedures as for adult participants, and no information that could identify a minor to a parent, guardian, or community member (including whether they disclosed personal experience of abortion) was shared outside the research team.

### Data analysis

Thematic analysis followed Braun and Clarke's six-phase framework: familiarisation, generating initial codes, searching for themes, reviewing themes, defining and naming themes, and producing the report. Both authors independently read and coded a subset of transcripts inductively, generating codes grounded in participants’ own language (e.g., “fear of the nurses,” “high costs,” “shame experienced by the family,” “side effects of the pill”). Codes were compared, discrepancies resolved through discussion until consensus was reached, and the coding frame was then applied to the remaining transcripts by authors.Where relevant, resulting themes were subsequently considered in relation to the constructs of the Health Belief Model (perceived susceptibility, perceived severity, perceived benefits, perceived barriers, cues to action, and self-efficacy), as reflected in the Results and Discussion section below. Illustrative participant quotations are used throughout to support the themes identified.

### Validity and trustworthiness

Trustworthiness was assessed against Lincoln and Guba's four criteria: credibility, transferability, dependability, and confirmability (12). Credibility was supported through prolonged engagement with participants, use of participant quotations to substantiate interpretive claims, and. Transferability was supported through detailed description of the study setting, participants, and context to allow readers to judge the applicability of findings to comparable settings. Dependability was supported through use of a standardised interview guide administered under comparable conditions across all interviews, and through maintenance of an audit trail of coding decisions. Confirmability was supported by deriving themes systematically from participant data and by having the coding and theme structure reviewed by the authors.

## Results and discussion

The four themes are presented below with illustrative participant quotations, followed in each case by discussion of how the theme relates to existing literature and to the Health Belief Model.

### Theme 1: perceived prevalence of unsafe abortion

#### Participant accounts

Across occupations, education levels, and communities, participants consistently described unsafe abortion as common:

“It is very common.” **— R1**

“Very common.” **— R14**

“Most often.” **— R33**

#### Interpretation

The consistency of this perception across a diverse group of respondents suggests it reflects a shared social understanding within the district, even though, as a qualitative study, this research cannot estimate how common unsafe abortion actually is. National data provide some context for this perception: the Ghana Health Service attributes around 11% of maternal deaths to unsafe abortion, and the Upper East Regional Health Directorate reports that teenage pregnancies account for 15% of antenatal cases in the region (4). Earlier reporting on Bongo District similarly pointed to elevated rates of teenage pregnancy and unsafe abortion, suggesting this is a long-standing rather than emerging concern.

One notable divergence emerged between community members and a healthcare provider: R3, a nurse, considered unsafe abortion “not common,” a view likely shaped by reliance on clinical records that capture only cases presenting with complications severe enough to require formal care. Because most unsafe abortions handled through informal channels are never recorded institutionally, such administrative data likely underestimate actual incidence; qualitative accounts from within the community may better reflect the practice as it is experienced locally (13).

Within the Health Belief Model, perceived prevalence would typically be expected to function as a cue to action, prompting protective behaviour. In Bongo District, however, participants’ accounts suggest the opposite dynamic may be occurring: the sense that unsafe abortion is widespread appears, if anything, to normalise recourse to informal providers and may reduce the perceived threat associated with them, rather than increasing motivation to seek safer alternatives.

### Theme 2: contraceptive knowledge, barriers, and the pathway to unsafe abortion

#### Participant accounts — knowledge

Many participants could name contraceptive methods (injectables, oral contraceptives, condoms, Depo-Provera, emergency contraception), indicating a degree of nominal awareness consistent with existing family-planning outreach in the district. This awareness, however, did not extend reliably to correct use, contraindications, or mechanism of action:

“No knowledge on family planning.” **— R2**

“Less knowledgeable.” **— R8**

“Ignorance of becoming pregnant.” **— R33**

These accounts echo prior research on adolescent reproductive health knowledge in northern Ghana (14). Within the Health Belief Model, meaningful engagement with a protective behaviour requires an accurate understanding of its benefits; where knowledge of a method's existence is not matched by understanding of how it works, that gap may be filled by misinformation rather than by informed decision-making.

#### Participant accounts — barriers

Fear of side effects was the most frequently cited barrier to contraceptive use, raised independently by more than fifteen participants:

“Fear of side effects.” **— R5, R6, R9, R10, R11**

“Some have the belief that it causes infertility.” **— R23**

“Myths and misconceptions.” **— R12**

Concerns about infertility, weight gain, hormonal changes, and physical harm, while not medically substantiated, functioned as genuine perceived barriers shaping behaviour, consistent with the Health Belief Model's emphasis on perceived (rather than objective) barriers as drivers of decision-making (15). This is consistent with prior Ghanaian research identifying misconceptions about side effects as a leading reason for contraceptive non-use among young women in rural areas (16, 17).

Relational and social barriers were also prominent, including anticipated judgment and partner disapproval:

“They feel shy to patronage family planning in the health facility.” **— R13**

“Disagreement from husbands.” **— R16**

“People would think I am promiscuous just because I want to be on any form of contraception.” **— R22**

Participants described not only general disapproval from male partners but specific fears of abandonment or verbal abuse when raising contraception. Where gender norms position men as the primary authority on fertility decisions, contraceptive discussion between partners becomes difficult regardless of a woman's own knowledge or intentions (16, 18). Prior research similarly identifies negative partner attitudes as a major, and frequently unaddressed, driver of contraceptive non-use in the region (19).

#### Participant accounts — pathway to unsafe abortion

Participants described the connection between contraceptive non-use and unsafe abortion directly:

“If they don't use contraceptives, they will get an unwanted pregnancy which leads to unsafe abortion.” **— R24**

“Not using contraceptives leads to unwanted pregnancy and eventually unsafe abortion.” **— R13**

“When preventive measures are not used and one becomes pregnant, because they don't want people to know about it, they choose the unsafe methods to keep it a secret.” **— R10**

This sequence, from non-use to unintended pregnancy to unsafe abortion, mirrors pathways documented elsewhere in sub-Saharan Africa (20). What the Bongo District accounts add is the apparent role of stigma in foreclosing intermediate options: participants suggest that it is not unintended pregnancy itself, but the perceived impossibility of confidential disclosure or support, that drives women toward unsafe procedures. Within the Health Belief Model, this suggests that the immediate, socially certain consequences of disclosure (family shame, expulsion from school, partner abandonment) may be perceived as more severe than the physical risks of an unsafe procedure, particularly in the absence of a trusted support network or a health system perceived as safe and confidential (16, 21).

### Theme 3: health-system access as a structural barrier

#### Participant accounts

Four recurring, mutually reinforcing barriers emerged: distance, cost, negative provider interactions, and confidentiality concerns.

##### Distance

For women in remote communities, distance from health facilities was described as prohibitive rather than merely inconvenient:

“It is difficult. Most houses are not close to the clinic.” **— R10**

“Very difficult because she will be referred to the regional hospital.” **— R31**

Referral to Bolgatanga Regional Hospital, in a separate municipality, involves transport costs that many participants could not afford. Physical distance has similarly been identified as a barrier to abortion care elsewhere in northern Ghana and across sub-Saharan Africa more broadly.

##### Cost

Cost was the most consistently cited barrier across communities and occupational groups:

“Expensive.” **— R21, R27**

“Most of these women are not working so lack funding.” **— R4**

“The cost of drugs is less compared to the hospital.” **— R4**

“It is less expensive and done secretly.” **— R21**

These accounts do not suggest a lack of awareness that formal care is clinically safer; rather, they describe an inability to act on that awareness because of cost, alongside a recognition that informal care also avoids the need for a public transaction. Prior work on the economics of unsafe abortion in sub-Saharan Africa similarly identifies price as a lever that demand-side subsidies or free provision could address (22).

##### Provider attitudes

Providers were frequently described as unwelcoming or judgmental toward young, unmarried women:

“Why would any young woman want to go there when some of these nurses are going to subject you to insults and also be judgmental?” **— R1**

“They behave harshly towards them.” **— R11**

“They are rude to them.” **— R21**

“After you leave, they will start talking about you and even be mentioning names, forgetting it is a public place.” **— R33**

Provider stigma as a structural barrier to reproductive healthcare is well documented in Ghana and across sub-Saharan Africa (23–25). Within the Health Belief Model, anticipated provider judgment functions as a perceived barrier that can deter care-seeking before a woman ever reaches a facility (26). Addressing this barrier has direct implications for youth-friendly service delivery: it points toward a need for provider training in respectful, non-judgmental, confidential care for young and unmarried women, alongside clearer institutional guidance on the legal scope of abortion services, since provider misunderstanding of the law may compound outright unwillingness to provide care.

##### Confidentiality

Because health workers and residents often belong to the same social networks, facility visits were not perceived as private:

“It is common for some of the sick people in the hospital to know the person.” **— R24**

“Because someone at the hospital might know me.” **— R10**

Structural stigma, in Hatzenbuehler's sense (27), can arise from institutional and social conditions without requiring hostile intent on the part of any individual provider. In a setting where facility and community networks overlap, information may travel regardless of formal confidentiality policy, making informal providers, embedded in the same networks but operating under different social expectations, appear more discreet by comparison (34, 35).

### Theme 4: socio-cultural and economic determinants

#### Stigma and family honour

Participants described unintended pregnancy as a social, rather than purely medical, event:

“Because of social stigma people want to eliminate it.” **— R4**

“It causes people to hide the truth.” **— R8**

“I know a friend who went it for an abortion couple of months ago; and the kind of guilt and shame the family faced was unbearable. You know this friend of mine all along made us look like we were the black sheep in our families when one of our friends got pregnant a year ago.” **— R32**

Where reproductive behaviour is understood as a matter of collective family standing rather than individual choice, an unmarried pregnancy can be experienced as a threat to a family's honour, not only an individual's circumstance. This resonates with Goffman's concept of “spoiled identity” (28) and with prior Ghanaian and sub-Saharan African research describing how fear of discovery can outweigh awareness of physical risk in shaping abortion-related decisions (16, 29).

#### Informal providers as a structural response

Traditional healers and chemical sellers were frequently described as a first choice rather than a last resort:

“They sell herbs, mixtures and pills; they are cheap; they are closer; they keep things to themselves.” **— R9**

“They are cheap; they keep secrets; easy access to them.” **— R24**

Reported methods ranged from pharmaceuticals and herbal remedies to more dangerous mechanical methods. Participants’ accounts suggest that women who use informal providers are generally aware of the associated risks; their choice appears to reflect the absence of an accessible, affordable, and confidential formal alternative rather than an underestimation of danger. This is consistent with a social-ecological reading in which the informal sector responds to, rather than creates, unmet demand (30). On this reading, young women may turn to informal providers because they perceive them as cheaper, closer, and more discreet than formal services, even though these options carry serious health risks; this should not be read as suggesting that informal, unsafe methods are an acceptable or desirable option, but as pointing to the structural conditions that make the formal system difficult to access (31–33). A corresponding policy implication is that measures targeting informal providers directly (restrictions, awareness campaigns) are unlikely to be effective unless accompanied by parallel improvements in the affordability, accessibility, confidentiality, and non-judgmental character of formal care.

#### Perceived solutions

When asked what would need to change, participants offered specific, non-hierarchical recommendations:

“I think there should be public education on unsafe abortion.” **— R4**

“The cost should reduce.” **— R18**

“The kind of attitude some healthcare providers show to the clients is not the best we should be treated with respect with less expensive abortion services.” **— R21**

“Sometimes when you are left at the mercies of the nurses’ they choose to treat you anyhow because they know that they are the only ones to offer help and to me I think they sometimes take undue advantage of young women who want to go through an abortion especially with regards to the pricing of service cost.” **— R33**

Participants did not rank these priorities relative to one another, suggesting an intuitive understanding that unsafe abortion in the district results from multiple, simultaneously operating causes. This is consistent with public health guidance suggesting that reducing unsafe abortion requires concurrent action on affordability (demand side), availability (supply side), and the normative environment shaping decision-making, rather than single-lever interventions such as contraceptive education or facility upgrades alone.

### Limitations

This study has several limitations. Findings reflect the perspectives of 34 young women from five communities in Bongo District and are not intended to be statistically generalisable to the district or beyond. The sensitivity of the topic may have contributed to social desirability or recall bias; while efforts were made to build rapport and assure confidentiality, some under-reporting or reframing of experiences cannot be ruled out. The retrospective nature of interviews also means the timing and sequencing of events could not be independently verified. Where interpretation was provided for participants preferring Gurune (Frafra), some loss of nuance in translation and transcription is possible, although convergence of themes across participants and communities supports the trustworthiness of the findings presented. Finally, this study did not include healthcare providers, parents, male partners, traditional healers, chemical sellers, or district health officials, all of whom are central to the dynamics discussed here; their perspectives represent an important direction for future research.

## Conclusion

Participants’ accounts suggest that unsafe abortion among young women in Bongo District reflects the interaction of structural, financial, and socio-cultural barriers rather than a simple lack of contraceptive knowledge. Most participants were aware, at least nominally, of contraceptive methods and of the risks of unsafe abortion, yet many nonetheless perceived traditional healers and chemical sellers as more accessible, affordable, and discreet than the formal health system. This study's main contribution is to show, from the perspective of young women themselves, how the perceived normalisation of unsafe abortion, financial and geographic barriers to formal care, provider attitudes, and fear of social consequences combine to shape this outcome. District health authorities may wish to consider fee waivers or subsidies for contraceptive and post-abortion care services, permanent structures for CHPS compounds lacking them, pre- and in-service provider training in youth-friendly and non-judgmental care, and community-based reproductive health education delivered through trusted local channels. Future mixed-methods or longitudinal research would help quantify the scale of unsafe abortion in the district and evaluate the effectiveness of such interventions.

## Data Availability

The raw data supporting the conclusions of this article will be made available by the authors, without undue reservation.
